# Neurocognition in adults with intracranial tumors: does location really matter?

**DOI:** 10.1007/s11060-022-04181-7

**Published:** 2022-11-08

**Authors:** Charlotte Sleurs, Catharina M. L. Zegers, Inge Compter, Jeanette Dijkstra, Monique H. M. E. Anten, Alida A. Postma, Olaf E. M. G. Schijns, Ann Hoeben, Margriet M. Sitskoorn, Wouter De Baene, Laurien De Roeck, Stefan Sunaert, Wouter Van Elmpt, Maarten Lambrecht, Daniëlle B. P. Eekers

**Affiliations:** 1 Department of Cognitive Neuropsychology, Tilburg University, Tilburg, The Netherlands; 2 Department of Oncology, KU Leuven, Leuven, Belgium; 3 Department of Radiation Oncology (Maastro), GROW School for Oncology and Reproduction, Maastricht University Medical Center+, Maastricht, The Netherlands; 4 Department of Medical Psychology, Maastricht University Medical Center+, MHeNs School for Mental Health and Neuroscience, Maastricht, The Netherlands; 5 Department of Neurology, Maastricht University Medical Center+, Maastricht, The Netherlands; 6 Department of Radiology & Nuclear Medicine, Maastricht University Medical Center+, MHeNs School for Mental Health and Neuroscience, Maastricht, The Netherlands; 7 Department of Neurosurgery, Maastricht University Medical Center+, MHeNs School for Mental Health and Neuroscience, Maastricht, The Netherlands; 8 Division of Medical Oncology, Department of Internal Medicine, GROW-School of Oncology and Developmental Biology, Maastricht University Medical Center, Maastricht, the Netherlands; 9 Department of Imaging and Pathology, KU Leuven, Leuven, Belgium

**Keywords:** Lesion-symptom mapping, MRI, Neurocognition, Neuro-oncology, Intracranial tumors

## Abstract

**Objective:**

As preservation of cognitive functioning increasingly becomes important in the light of ameliorated survival after intracranial tumor treatments, identification of eloquent brain areas would enable optimization of these treatments.

**Methods:**

This cohort study enrolled adult intracranial tumor patients who received neuropsychological assessments pre-irradiation, estimating processing speed, verbal fluency and memory. Anatomical magnetic resonance imaging scans were used for multivariate voxel-wise lesion-symptom predictions of the test scores (corrected for age, gender, educational level, histological subtype, surgery, and tumor volume). Potential effects of histological and molecular subtype and corresponding WHO grades on the risk of cognitive impairment were investigated using Chi square tests. P-values were adjusted for multiple comparisons (p < .001 and p < .05 for voxel- and cluster-level, resp.).

**Results:**

A cohort of 179 intracranial tumor patients was included [aged 19–85 years, median age (SD) = 58.46 (14.62), 50% females]. In this cohort, test-specific impairment was detected in 20–30% of patients. Higher WHO grade was associated with lower processing speed, cognitive flexibility and delayed memory in gliomas, while no acute surgery-effects were found. No grading, nor surgery effects were found in meningiomas. The voxel-wise analyses showed that tumor locations in left temporal areas and right temporo-parietal areas were related to verbal memory and processing speed, respectively.

**Interpretation:**

Patients with intracranial tumors affecting the left temporal areas and right temporo-parietal areas might specifically be vulnerable for lower verbal memory and processing speed. These specific patients at-risk might benefit from early-stage interventions. Furthermore, based on future validation studies, imaging-informed surgical and radiotherapy planning could further be improved.

**Supplementary Information:**

The online version contains supplementary material available at 10.1007/s11060-022-04181-7.

## Introduction

Treatments for intracranial tumors have tremendously evolved throughout the last decades [1, 2]. Although survival rates have been rising gradually, many survivors experience medical and psychological sequelae in their daily life [3]. One topic that has increasingly received attention, is the risk for neurocognitive decline in this population [4, 5]. However, the prevalence rates of this problem is diagnosis-specific and has been reported very inconsistently to date, and individual risk profiling consequently remains an important goal for the scientific neuro-oncological community [6]. A few steps in this direction have been taken, including investigations of individual risk factors such as fatigue and emotional difficulties [7], cognitive reserve and education level [7, 8], genetic subtypes [9], molecular tumor profiling[10], as well as detailed treatment characteristics (e.g. cranial radiation dosimetry, chemotherapeutic agents, neurosurgical strategy including fiber tract analysis and neuromonitoring) [4, 5, 11].

Although the number of studies on potential individual risk factors for cognitive decline are growing, the predictive value of neuroimaging features remains inconclusive [12, 13]. Multiple imaging studies have evidenced radiation-induced neurological damage which can be related to neurocognitive decline in multiple domains [14]. However, it remains uncertain to which extent the tumor itself (its focal as well as compressing or infiltrative effect) plays a role in baseline cognitive performance. Imaging-based predictions of cognitive functioning before the start of (radiation) therapy have only received limited attention [15]. Hence, the question arises to which extent treatment should be adapted to the tumor location, sparing functionally crucial areas for cognitive outcomes. To address regional sensitivity of tumoral damage and its functional impact in detail, lesion-behavior analyses have been accumulating [16]. Initially, individual cases and clinical observations helped to address focused region-based lesion-behavior investigations, for instance suggesting the Broca area to be an important functional hub for speech production [17]. However, since the 20th century, the integration of clinical neuroradiological knowledge and neurological observations has gradually provided more insights into structure–function relationships. Furthermore, the neuroscientific community is shifting towards pre-intervention cerebral network analyses [18]. To investigate functional brain hubs, we can nowadays profit from more advanced imaging analysis techniques. For instance, analyses such as voxel-based approaches have been proposed and optimized [19, 20].

In addition, neurocognitive test assessments consisting of multiple assessments can provide a more comprehensive overview of the complex individual cognitive profile of patients compared to single neuropsychological tasks.

The combination of neuropsychological assessment and advanced neuroimaging techniques, can result in improved detection and individual neuropsychological risk profiling. In neuro-oncology specifically, existing voxel-based lesion-symptom mapping studies have mainly focused on glioma patients so far [21–24], with lower cognitive scores in case of lesions in middle temporal gyrus [23, 25–27], with possibly left hemisphere dominance for language-dependent tasks [23, 26] and right dominance for visual attention or processing speed [22]. However, baseline performance of the complete population of intracranial tumors has not yet received attention in this field to date. Furthermore, the existing findings remain very inconsistent, with relatively small cohorts and univariate imaging statistics [21, 22, 24, 28]. Therefore, an investigation of a large neuro-oncological cohort covering all intracranial tumor types was performed in this study [29].

## Materials and methods

### Participants

Adult intracranial tumor patients aged between 18 and 80 years old who were scheduled to receive cranial radiotherapy, received cognitive testing (2–6 weeks post-surgery) as part of their standard care between April 2019 and March 2022. Diagnoses consisted of adult intracranial tumors (i.e. meningiomas, gliomas, vestibular schwannomas, pituitary adenomas, craniopharyngiomas and others). Exclusion criteria consisted of being unable to perform the cognitive tests (e.g. due to malaise, substantial hearing or vision loss, or chronic fatigue), an MRI-scan of insufficient quality at baseline.

### Materials

Each patient underwent a baseline neuronavigation MRI procedure for radiotherapy planning. 3 T MR scans (1 mm slice thickness) were acquired in standard sagittal T2-weighted FLAIR scans and a gadolinium (Gadovist^©^ 1.0 mmol/ml 0.1 mL/kg bodyweight) contrast-enhanced axial T1-weighted sequence (T1w). A baseline pre-radiation cognitive test assessment was additionally acquired. This test battery included the Hopkins Verbal Learning Test (HVLT), the Controlled Oral Word-Association Test (COWA) and the Trail Making Test, taking about 30 min in total. Each cognitive test score (HVLT learning phase, HVLT delayed recall, COWA phonemic fluency, TMT A, TMT B) was normalized to a z-score using test-specific international normative data [30–32]. Based on these tests, we estimated immediate and delayed recall, phonemic fluency, processing speed and cognitive flexibility, respectively. In addition, education level was requested and graded according to the Dutch Verhage scale (1964)[33] (i.e. level 1 = less than 6 years of primary education, up to level 7 presenting a university degree). Data including demographic characteristics (gender and age) and treatment-related characteristics (histological tumor subtype, type and location of surgery) were recorded as potential covariates of interest.

### Image processing

Pre-radiation gross tumor volumes (GTVs) (i.e. complete tumor volume or post-surgical residual tumor tissue + resection cavity) were delineated by an experienced radiation neuro-oncologist based on the anatomical MRI scans, which were co-registered with a CT scan for radiotherapy treatment planning. These delineations were performed according to (inter)national guidelines and double-checked by a second rater [34, 35]. For statistical analyses, CT scans were then (reversely) linearly registered (rigid transformation) to the post-contrast T1w MRI scan. The same transformation was applied to the GTVs (with nearest neighbor interpolation). Second, after skull stripping, the brains on pre-contrast T1w MRI scans were non-linearly registered (rigid, affine and deformable transformation) to a population-based brain T1w MRI template (i.e. ICBM-MNI). Again, this transformation was applied to the GTVs (with nearest neighbor interpolation). All registrations were performed using Advanced Normalization Tools [36]. Once all GTVs were in template space, voxel-wise statistics were performed.

### Statistical analyses

First, frequencies of impairment per test are reported. Impairment was defined as $\ge$ 2 standard deviations below the normative mean for each test separately [37]. Frequencies of impairment on each task were compared between the different histological subtypes (a), as well as between WHO grades (b) and surgery subgroups (no surgery, biopsy, resection) (c), using likelihood chi-square tests (G^2^) (for which (b) and (c) were only performed within the glioma and meningioma subgroups). Second, support vector regression voxel-based lesion‐symptom mapping was implemented. This model predicted each (normalized) cognitive test score based on lesion location in each voxel, after regressing out lesion volume of both the behavioral data and lesion data. Covariates in the model predicting test scores included age at assessment, gender and education. The models were repeated to correct for potential tumor- and surgery-specific effects by including histological tumor subtype and type of surgery (no, biopsy, resection) as additional covariates. Permutation testing (with 1000 permutations) was performed with the level of significance set at p < 0.001 at voxel-level, and at p < 0.05 at cluster‐level. Only voxels occurring in at least 10 patients were included to reach sufficient lesion affection and remove spurious voxels from analyses. These analyses were conducted using the MATLAB-based multivariate lesion symptom mapping toolbox [38].

## Results

**Fig. 1:**
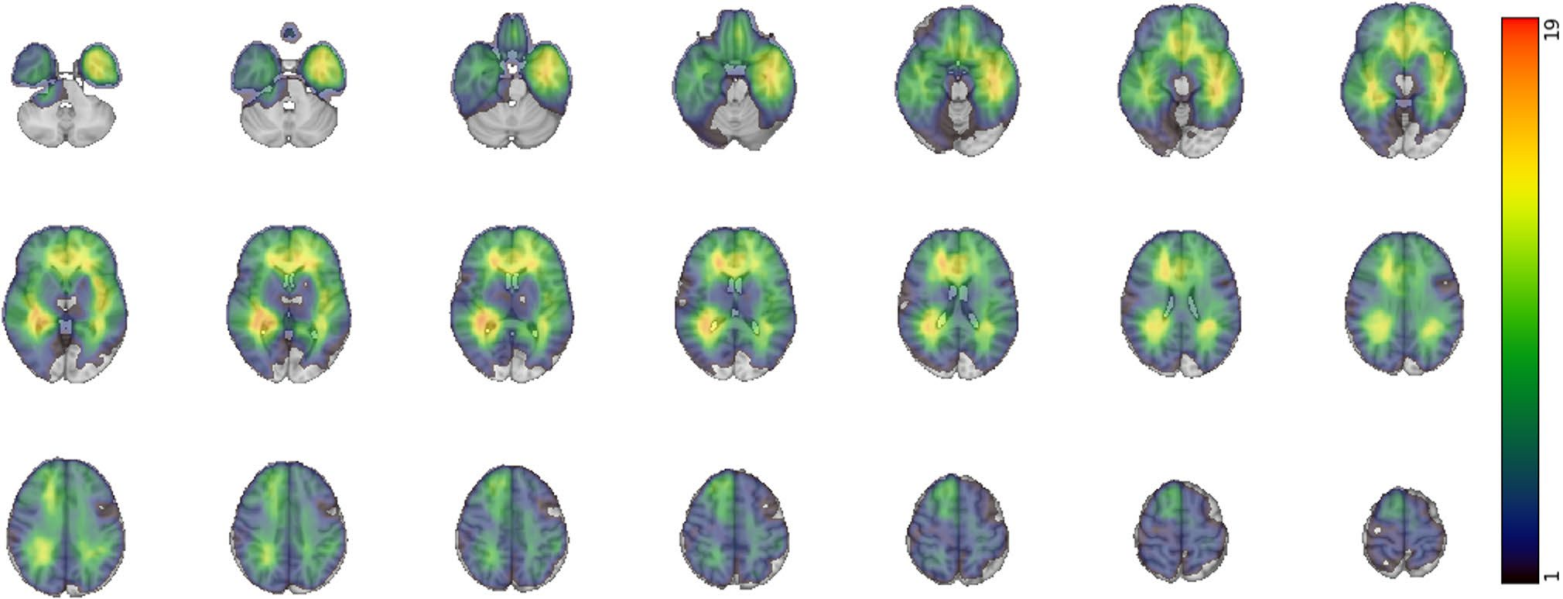
Voxel-wise heatmap of lesion presence *Note*. The maximum amount of overlapping lesions was n = 21. The heatmap shows yellow to red areas where lesions were mostly occurring (median = 5). The majority of lesions affected the left inferior temporal, left insular, right superior temporal and anterior cingulate cortex. Images are shown according to radiological convention

In total, 179 patients were included in this study (for demographic information, see Table 1). The majority of patients were diagnosed with gliomas (n = 126), followed by meningiomas (n = 28), vestibular schwannomas (n = 9), pituitary adenomas (n = 9), craniopharyngiomas (n = 4) and others (n = 4). A heatmap of voxel-wise lesion prevalence is presented in Fig. 1. Tumor-specific heatmaps (and low- vs. high-grade glioma heatmaps) are available in Supplementary Figs. 1–5. Lesions were most often detected in (or surrounding) the temporal and frontal lobes, more specifically involving the left inferior temporal gyrus, right superior temporal gyrus and right anterior cingulate cortex (Fig. 1). These locations mainly match with the gliomas and meningioma subgroups, given that these cover the majority of patients.

**Table 1:** Descriptive characteristics of patient population (n = 179)

Characteristics	N (n total = 179)		Percentage	
Demographic				
Median age in years (SD)	58.46 (14.62)			
Gender: Females	90/179		50.28%	
Tumor location				
Central region	13/179		7.26%	
Cerebellar tumor	18/179		10.05%	
Frontal(-parietal) tumor	57/179 (2/179)		31.84% (1.12%)	
Occipital tumor	5/179		2.79%	
Parietal(-occipital) tumor	29/179 (5/179)		16.20% (2.79%)	
Temporal tumor	50/179		27.93%	
Involved hemisphere^a^				
Left-sided	72/179		40.22%	
Right-sided	93/179		51.96%	
Bilateral	14/179		7.82%	
Surgery				
No surgery	35/179		19.55%	
Biopsy	41/179		22.91%	
Resection	103/179		57.54%	
Tumor histology				
Gliomas	126/179		70.39%	
Meningiomas	28/179		15.64%	
Vestibular schwannomas	9/179		5.03%	
Pituitary adenomas	9/179		5.03%	
Craniopharyngiomas	4/179		2.23%	
Other	4/179		2.23%	
Cognitive impairment^b^				
No impairment	79/179		44.13%	
$\ge$ 1/5 test scores	100/179		55.87%	
$\ge$ 2/5 test scores	68/179		37.99%	
$\ge$ 3/5 test scores	30/179		16.76%	
WHO grade^a^	Gliomas	Meningiomas	Gliomas	Meningiomas
1	3/124	15/28	2.42%	53.57%
2	35/124	11/28	28.23%	39.29%
3	22/124	2/28	17.74%	7.14%
4	64/124	NA	51.61%	NA

*NA*   not applicable, *N*   number

^a^Hemispheric locations and WHO grades were only clinically defined for gliomas and meningiomas. Grades were available for 150 of these patients in total

^b^Cognitive impairment was defined as $\ge$ 2 standard deviations below the normative mean

Cognitive impairment was detected for immediate recall in 21.78% (n = 39/179), delayed recall in 26.28% (n = 46/175), processing speed in 26.96% (n = 48/178), cognitive flexibility in 31.79% (n = 55/173) and phonemic fluency in 20.22% (n = 36/178) of all cases. The prevalence of impairment only significantly differed between tumor subtypes on the processing speed task, with glioma patients and vestibular schwannoma patients showing the highest prevalence with 32.25% (n = 40/124) of impairment in gliomas, 14.28% (n = 4/28) in meningiomas, 33.33% (n = 3/9) in vestibular schwannomas, 25% (n = 1/4) in craniopharyngiomas, 0% (n = 0/9) in pituitary adenomas or other diagnoses; *G*^*2*^ = 12.65, *p* = 0.027). Regarding WHO grades within the meningioma subgroup, no significant association between grade and risk of impairment was found (meningiomas: G^2^ = 0.76, p = 0.69; G^2^ = 1.29, p = 0.53; G^*2*^ = 0.76, p = 0.69; G^2^ = 4.5, p = 0.105; G^2^ = 2.73, p = 0.26; for immediate recall, delayed recall, processing speed, cognitive flexibility and phonemic fluency, respectively).

Regarding WHO grade in glioma patients, significantly lower scores were found in the WHO 4 subgroup for processing speed (TMT A) (33.33% in WHO 1, 14.70% in WHO 2, 13.64% in WHO 3, and 47.61% in WHO 4; G^2^ = 15.96, p = 0.001), as well as in cognitive flexibility (TMT B) (66.66% in WHO 1, 20.59% in WHO 2, 23.81% in WHO 3, and 50% in WHO 4; G^2^ = 11.25, p = 0.010) and in both WHO 3 and WHO 4 for delayed recall (HVLT B) (0% in WHO 1, 11.76% in WHO 2, 38.10% in WHO 3, and 32.79% in WHO 4; G^2^ = 8.83, p = 0.03). No grading effects were found for the other tasks of phonemic fluency (COWA), nor immediate recall (HVLT A) (G^2^ = 3.75, p = 0.29*; *G^2^ = 5.22, p = 0.156, resp.). The exact numbers of cases can be found in supplementary materials (Suppl. Table 1).

Regarding the different neurosurgerical procedures, no group differences (between “no surgery” versus “biopsy” versus “resection”) in impairment frequencies were found within the glioma, nor within the meningioma subgroup (Suppl. Tables 2 and 3).

**Fig. 2:**
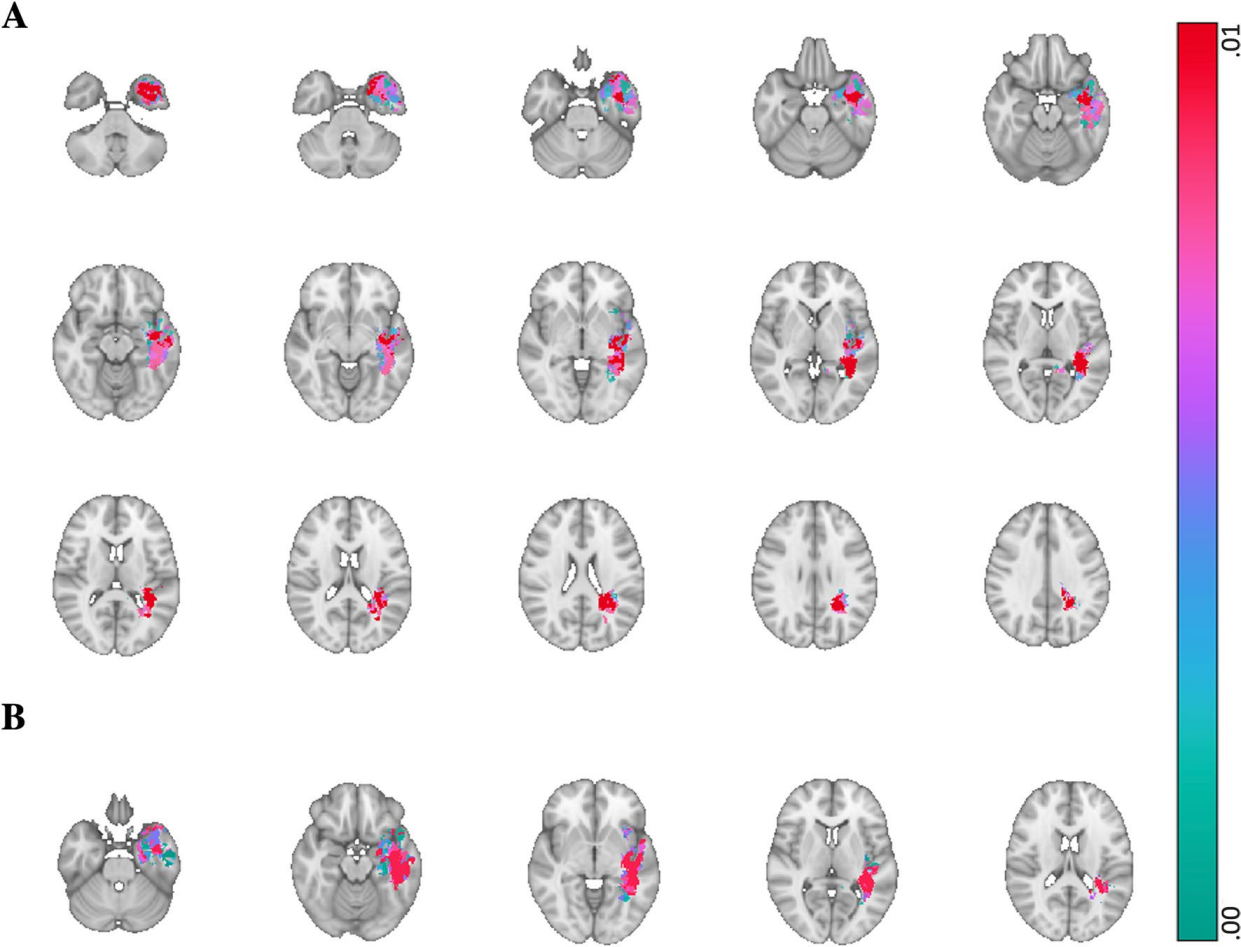
Voxel-wise p-map for predictions of verbal memory scores *Note*. This p-map shows green to red voxels where lesions were significantly associated with immediate and delayed verbal memory scores (as estimated with HVLT-A and HVLT-B). Panel A shows the regions associated with immediate verbal memory. Panel B shows the regions associated with delayed verbal memory. Red indicates the clusters that were significant at cluster level (affecting the left superior temporal gyrus and temporal pole). Images are shown according to radiological convention

With regard to verbal memory, immediate and delayed recall (HVLT A, B) were significantly associated with lesions affecting the left temporal lobe (i.e. involving the superior gyrus and temporal pole) (see Fig. 2). More specifically, the significant lesion cluster predicting delayed recall was most closely located to the left hippocampus, while the significant cluster associated with immediate recall was located more laterally affecting the parahippocampal area and insular region.

**Fig. 3:**
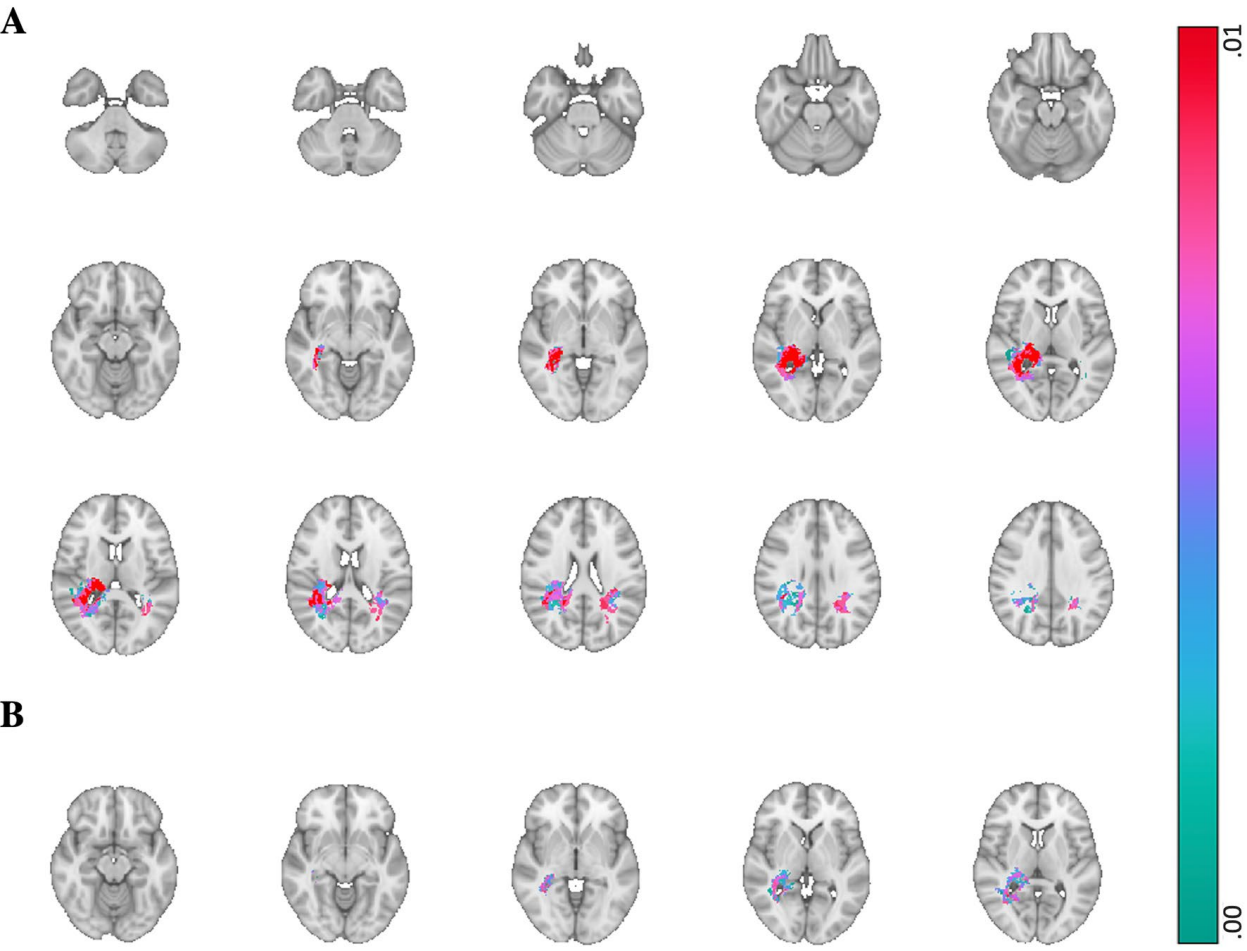
Voxel-wise p-map for predictions of processing speed and cognitive flexibility performance *Note*. This p-map shows green to red voxels where lesions were significantly associated with processing speed scores (as estimated with TMT-A) and cognitive flexibility scores (as estimated with TMT-B), in panels A and B, respectively. Red indicates the clusters that were significant at cluster level. In panel A, these clusters were located surrounding the right temporo-parietal junction, extending to the dentate gyrus of the hippocampus and fornix. In panel B, no voxels were significant at cluster level. Images are shown according to radiological convention

For processing speed (TMT A), a significant cluster was encountered surrounding the right temporo-parietal junction, extending to the dentate gyrus of the hippocampus and fornix (see Fig. 3). A smaller significant area associated with cognitive flexibility (TMT B) was largely overlapping with the cluster associated with processing speed. However, this area did not reach significance at cluster-level.

Regarding verbal fluency, a few voxels in the left superior temporal gyrus were significantly associated with fluency scores. However, these associations were not significant at cluster-level. Finally, all significant clusters were reproduced when tumor subtype and resection subtype were additionally included as covariates.

## Discussion

This cohort study of patients with intracranial tumors showed cognitive impairments in about 20–30% of cases. More specifically, tumor histology was significantly associated with impairment on the processing speed task. Grading analyses further showed an increased risk of impairment for high-grade compared to low-grade gliomas in processing speed, cognitive flexibility and delayed memory, while no acute surgery-effects were found. Based on our multivariate voxel-wise analyses, tumor location was only significantly predictive of verbal memory and processing speed, involving the left superior temporal gyrus, temporal pole and (para-)hippocampus, and right temporo-parietal junction, hippocampal and fornix areas, respectively.

These findings are in line with evidence from functional MRI studies, showing activity in similar areas during memory [39] and visual processing or flexibility tasks [40, 41]. Although previous VLSM studies also mostly reported significant findings in temporal gyri [23, 25–27], the existing results remained inconsistent [21, 22, 24, 28]. This heterogeneity could partly be attributed to the use of different test materials across studies, but also to the different predominant lesion locations. More specifically, in our cohort, the glioma subtype appeared to be mainly located in left temporal and frontal areas. This predominancy is in concordance with the recent findings of Habets et al. (2019) [21]. In contrast to most VLSM studies, the current study allows to perform a comparison at a larger scale (between tumoral location, histological and grading effects) by covering the entire adult intracranial tumor population. The fact that cognitive impairment was only histology-related for processing speed, with glioma (and schwannoma, albeit based on a small cohort n = 9) patients being potentially at higher risk, confirms that these specific tumoral cells interact with brain areas associated with processing speed, while compression by meningiomas seem to have a smaller effect. In particular, we found associations between lesions affecting the right temporo-parietal area, hippocampal and fornix and lower processing speed scores. Not only might the tumoral cells interact differently, also the predominant locations (and related functional outcomes) of the different tumor types are different (as shown in the tumor-specific heatmaps). More specifically, significant clusters related to processing speed and cognitive flexibility are mainly overlapping with supratentorial locations of gliomas (not with the heatmaps of other subtypes), whereas the areas related to immediate and delayed verbal memory recall overlap with both glioma and meningioma locations (again not the other subtypes). This can suggest that mainly glioma and meningioma patients with tumors located in (or surrounding and hence affecting) the encountered cluster areas are specifically at risk for such specific cognitive problems in daily life. Moreover, cognitive tests such as HVLT and TMT can be particularly important to implement in clinical routine for these populations.

Although we did not encounter surgery-related associations with impairment frequencies, we need to keep in mind that the distribution regarding surgery was skewed, with the majority that underwent surgery (41 biopsy and 103 had resection). Furthermore, cognitive assessments took place only shortly after surgery (app. 2–6 weeks), so we can only conclude that there were no acute symptoms. Longer follow-up is required to assess if and how specific surgical techniques (biopsy and resection) might lead to certain cognitive sequelae at a later timepoint.

Besides the histological subtypes, also the aggressiveness of the tumor can differently affect healthy brain tissue. For instance, glioblastoma is known to invade and grow faster, which results in edema, a remarkable mass effect and intracranial pressure, leading to substantial damage to the healthy tissue [42]. More specifically, the brain network could reorganize more efficiently in case of slower tumoral growth patterns (e.g. low-grade gliomas) compared to the more aggressive tumor types (e.g. glioblastomas). Hence, in this study we additionally investigated the impact of WHO grade, which showed high-grade glioma patients indeed to be specifically at increased risk for impairment in processing speed, cognitive flexibility, and delayed memory. Although tumor locations of these two subgroups differed in our study, with high-grade gliomas occurring more often in the right hemisphere, and low-grade gliomas more often in the left hemisphere (see Appendix), the lesion-outcome locations do not exactly match with these grade-specific heatmaps. Hence, tumor grade appears to be an additional risk factor on top of the encountered task-specific lesion location, for gliomas specifically. Detailed analyses of tumoral molecular markers have previously also been associated with brain atrophy (e.g. IDH status [43], 1p/19q co-deletion and TERT promoter mutation [44, 45]) and cognitive performance (e.g. IDH-1 expression, CD3, ATRX, BDNF, EAAT1, GAT-3, SRF, NLGN3, CK2Beta and P-STAT5b, NLGN3 and CK2Beta [45]). Still, interactions between such detailed molecular features and lesion location-behavior relationships need further investigation in the future. In contrast to gliomas, we did not find grading effects for meningioma patients.

Although tumor-related factors can play an important role in neurocognitive outcomes of a patient, the final outcome of the patient is more complicated than tumor-related only. Neurodegenerative, connectome and metabolic changes are not only affected by the tumor or surgery, but also by patient-related factors such as age, cognitive reserve or education level, gender, and genetic factors [46], as well as additional treatments (including anti-epileptic drugs [47] and corticosteroid treatment and possible medical complications (including epileptic seizures [48]). Each of these components is intrinsically related to the tumor type, which can complicate the correct risk stratification for neurocognitive decline. Hence, heterogeneity of findings across VLSM studies can partly be explained by patient-related differences in the investigated samples, as well as in the statistical approach, either including covariates or not. In this study, covariates age, gender, education, tumor volume, histology/grade were all included in the full models. More large-scale studies applying models that can sufficiently explain the existing large variability in neuropsychological performance of neuro-oncological patients would be recommended. Furthermore, VLSM analyses focus on brain area-predicted outcomes from a localism perspective, while the neuroscientific field is moving towards connectomics [18]. Applying connectomic and network-based lesion symptom mapping imaging techniques (including resting state fMRI and diffusion-weighted MRI to estimate functional and structural brain networks, respectively) combined with daily life measures for neuro-oncological patients might accelerate building our knowledge on which brain connections to spare during surgery, irradiation, and to stimulate during interventions. Even more, given that brain reorganization, consequently functional hubs (and thus regional vulnerability) could depend on the subtype and aggressiveness of the tumor, network analyses could provide more insight into the differential and dynamic structure–function relationships in future studies.

Patients with gliomas or meningiomas affecting the abovementioned temporal and temporo-parietal areas could possibly benefit from early (i.e. from diagnosis onwards) onset neurorehabilitation interventions [49], with computerized interventions, transcortical magnetic stimulation [50], and psychopharmacology [51], of which each need further investigation for effectiveness. Interventions such as stimulation might also implement information from VLSM studies, with a potential focus on temporo-parietal areas for specific cognitive decline in verbal memory and attention.

Alongside interventional trials, at this point, prevention remains key. Both prevention and intervention which can spare neuropsychological functioning is important, as it is not only important for daily quality of life of the patient, but also for patients to understand the treatment, informed consent of the treatment and for treatment adherence [52, 53].

Some limitations and strengths need to be considered when interpreting the results. First, we need to mention that the majority of patients in this cohort were diagnosed with a glioma, and the remaining tumor types covered relatively small subgroups. Hence, the final results can be mainly driven by the glioma subgroup and its predominant locations. However, we note that there is a lack of research for the non-glioma populations, which is mainly due to their low prevalence rates. Therefore, we aimed to investigate all intracranial tumor types and to cover the entire brain tissue for brain-behavior analyses. Hence, the included lesions were defined as “affected brain tissue” more in general, which could include tumoral tissue, cavity as well as compression by the tumor. To account for this heterogeneity in lesions, analyses were maximally corrected for tumor histology subtype as well as surgery type. Second, as delineations were provided by different radiation oncologists in clinical care, inter-rater variability cannot be excluded. Still, each of these clinicians were trained according to a standardized protocol, and delineations were double-checked by a second colleague. Third, the scans were acquired on two different MRI scanners, which results in inter-scanner variability. Hence, the images were intensity-normalized before the registrations to the common template. Fourth, neuropsychological assessments were acquired by assessors trained by a neuropsychologist, but not necessarily blinded to the patient information. Fifth, specific test materials were selected according to the recommendations of the European Particle Network. We cannot exclude the possibility that our imaging-related findings are specific to the applied tests (i.e. learning of word lists in HVLT and sequential ordering of numbers in TMT-A), rather than generalizable to more general cognitive domains. For instance, one earlier study in patients with meningiomas showed frontal rather than temporal brain involvement in the computerized cognitive flexibility tasks that were used [54]. Relatedly, cognitive impairment was defined as deviating scores that exceed the cut-off of two standard deviations below the norm. By selecting this relatively stringent cut-off, we cannot exclude the possibility of daily life impact in patients who did not exceed this cut-off for the measured cognitive tests. Previous research often used less stringent cut-offs, which could have affected our results which could thus mainly focus on the most affected patients. Finally, some patients had a subtotal resection. In other words, some delineated volumes pre-RT consisted of both tumoral tissue and a resection cavity. In this study, the gross tumor volume, consisting of residual tumor (if any) and cavity, was used as volume of interest, as each voxel is non-healthy brain tissue (i.e. tumoral or resected tissue) and can therefore be involved in cognitive decline.

Regarding the strengths of this study, multiple tumor types were investigated in this study, covering all brain areas that are potentially important for cognitive outcomes. Most earlier studies only focused on glioma tumors only, while this study shows different heatmaps for each histological subtype, and functional brain clusters that could be important predictors for cognitive outcomes. Not only gliomas can affect these specific areas, but also meningiomas, and in case of larger tumors even other subtypes can lead to compression of these specific brain areas as well.

From a statistical point of view, a state-of-the art procedure of a support vector multivariate analysis was selected, as multiple voxels are considered at once, which reduces the number of applied tests, and inter-voxel relationships are considered. Furthermore, the criterion of ‘sufficient lesion affection’ was fulfilled since voxels were only included with the minimum of 10 patients having a lesion in that voxel [19]. In addition, tumor volume was incorporated as factor of interest for both the lesion (or voxel) locations as well as for the cognitive scores, as it was regressed out of both, as recommended for VLSM studies most recently [38]. Permutation testing was chosen with stringent voxel- and cluster-level significance levels to sufficiently correct for multiple testing [20]. This approach solves the issue of the unmet assumption of uneven distributions of voxel-values.

## Conclusion

In this cohort study, variable test-specific cognitive impairment was observed in about 20–30% of neuro-oncological patients, of which processing speed was specifically histology-related and mainly impaired in glioma and vestibular schwannoma patients. In the lesion-symptom mapping analyses, tumors affecting the left temporal areas and right temporo-parietal areas were related to verbal memory and processing speed, respectively. Both gliomas and meningiomas can occur within or compressing these specific areas, so they might benefit from early interventions, if the lesions specifically involve damage or compression of temporo-parietal areas. Even more, processing speed or flexibility might be crucial to assess in glioma patients, while verbal memory should be assessed in both glioma and meningioma subgroups, with high-grade glioma patients being most at risk. Future multi-diagnosis multivariate VLSM studies are required to confirm these findings.

## Supplementary Information

Below is the link to the electronic supplementary material.

Supplementary file1 (DOCX 5792 kb)

## Data Availability

Pseudonymized data can be shared upon request, with a data-transfer agreement and after approval by the local ethical committee.
