# Supplementary material for: Neurocognition in adults with intracranial tumors: does location really matter?
Source: J Neurooncol. 2022 Nov 8;160(3):619–29. doi: 10.1007/s11060-022-04181-7 (PMC9758085; doi:10.1007/s11060-022-04181-7)
Supplement: Supplementary file 1 — Supplementary file1 (DOCX 5792 kb) [file 11060_2022_4181_MOESM1_ESM.docx]

**Supplementary Materials**

Heatmaps per histological subtype


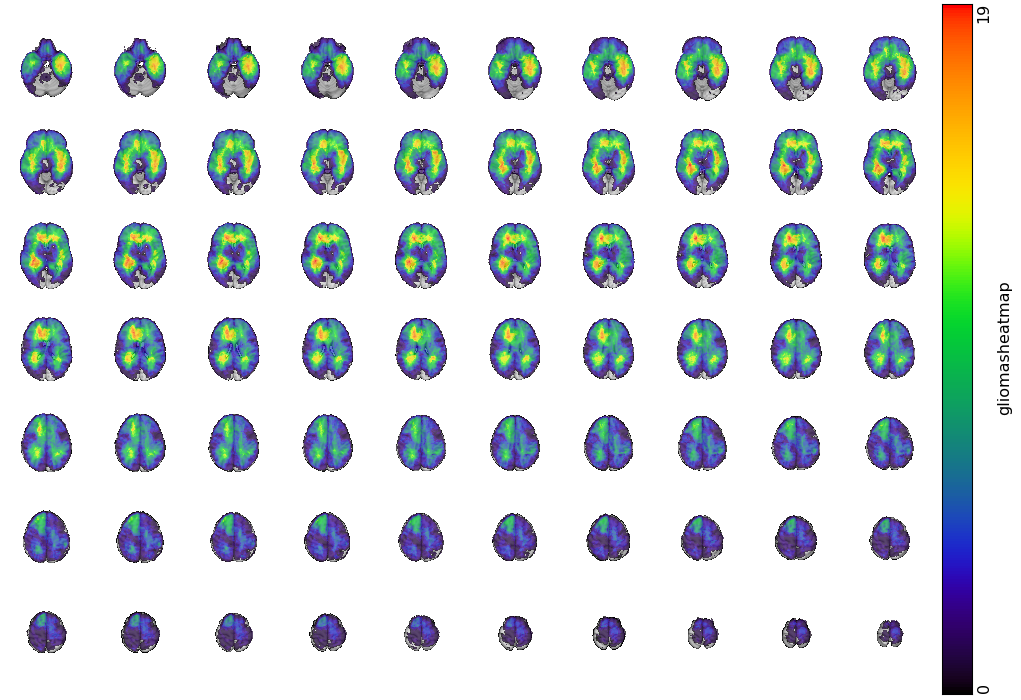

Supplementary Figure 1a. Overall glioma heatmap


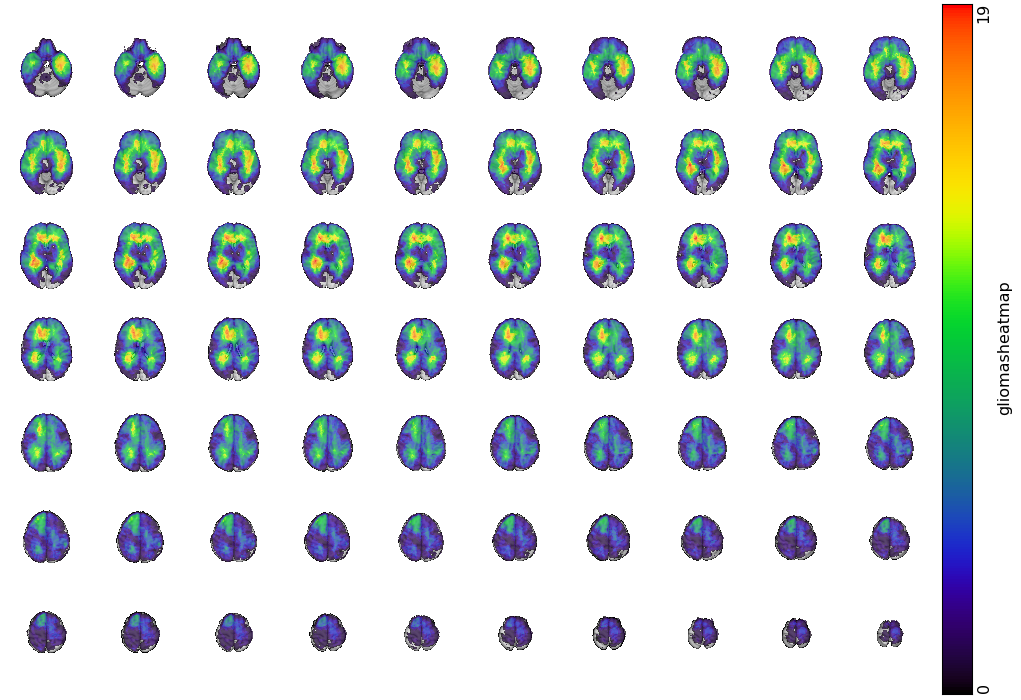


1 11


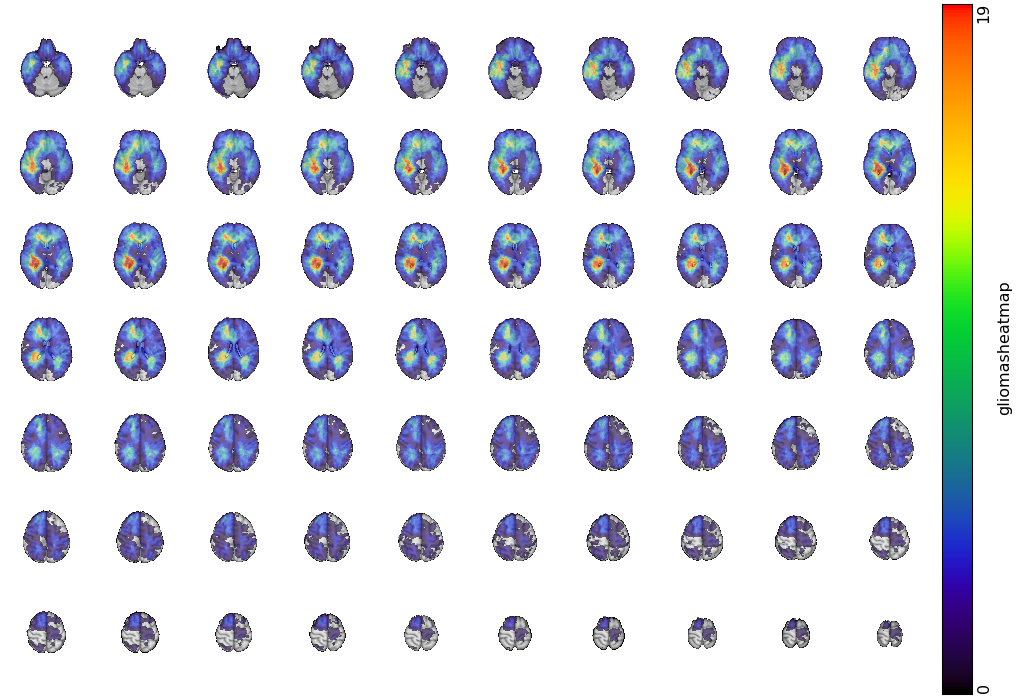


Supplementary Figure 1b. High-grade glioma heatmap


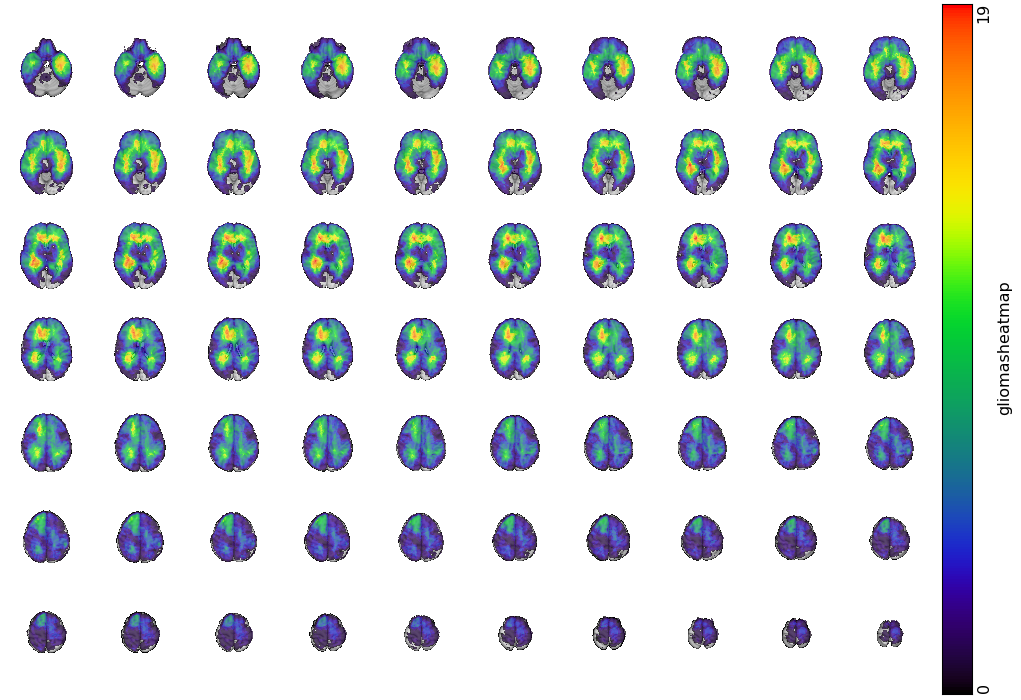


1 19


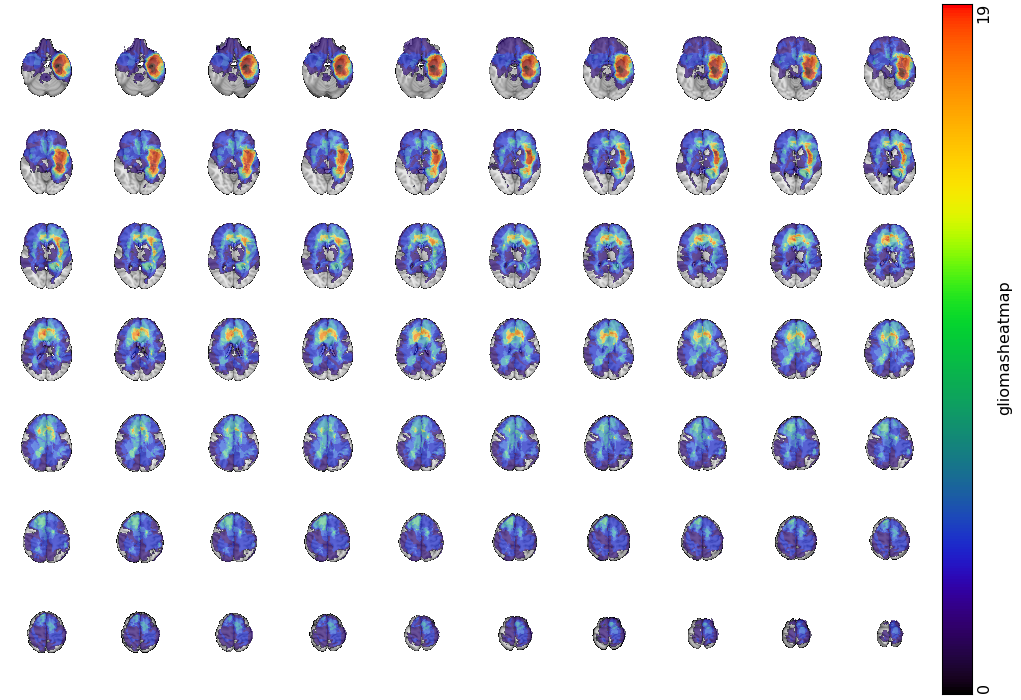
Supplementary Figure 1c. Low-grade glioma heatmap


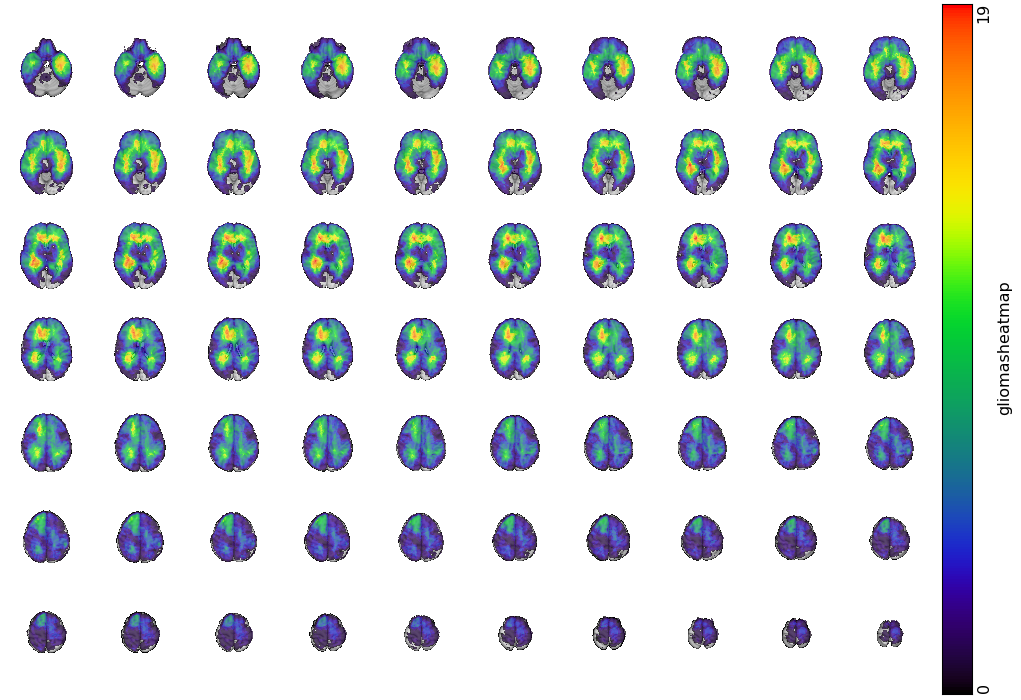


1 5


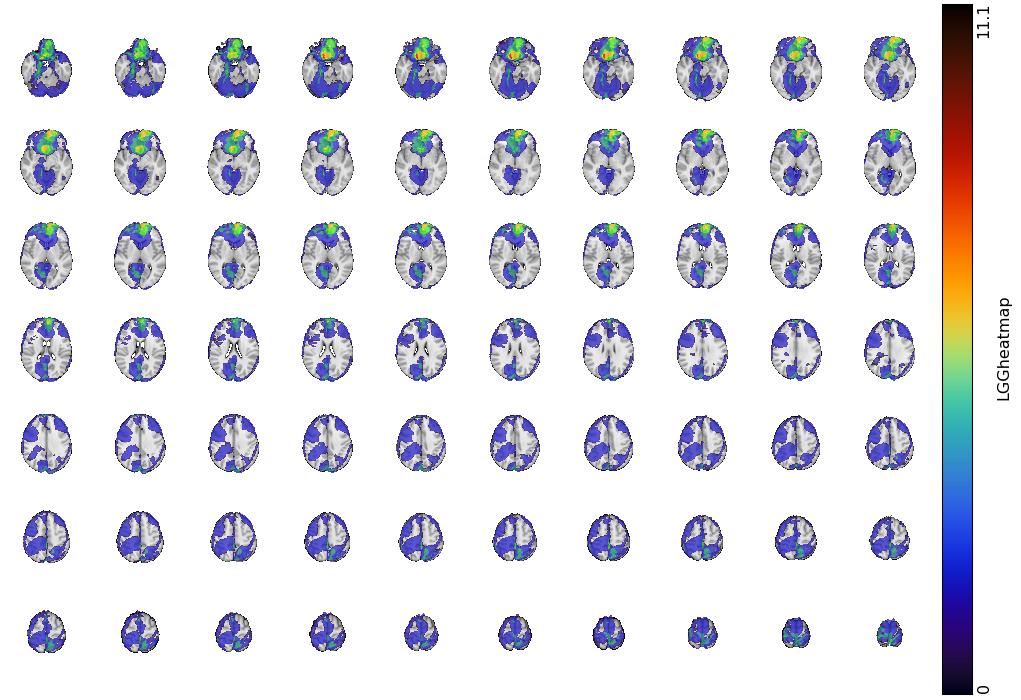


Supplementary Figure 2. Meningioma heatmap


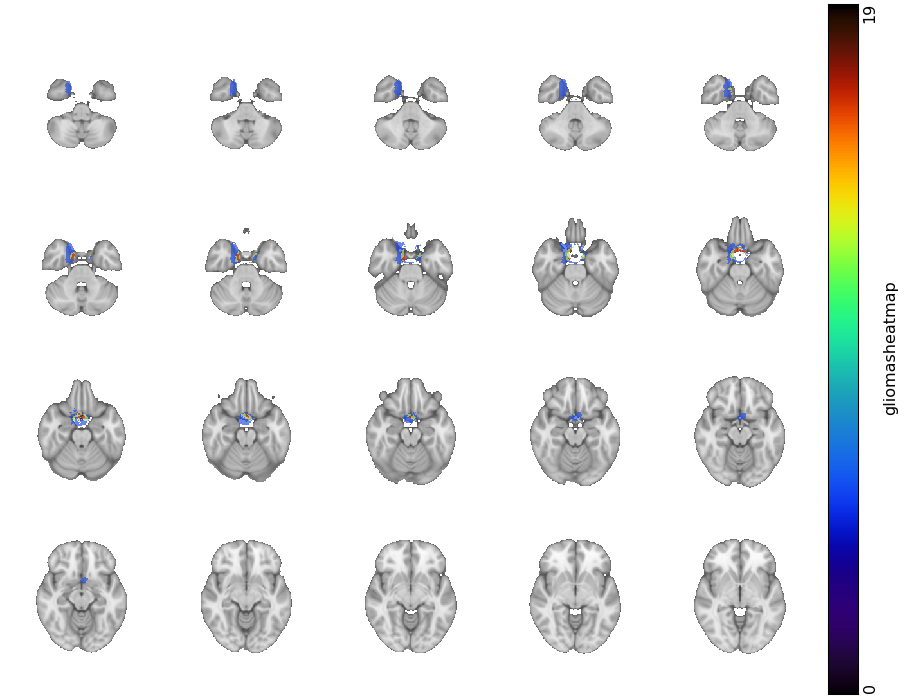


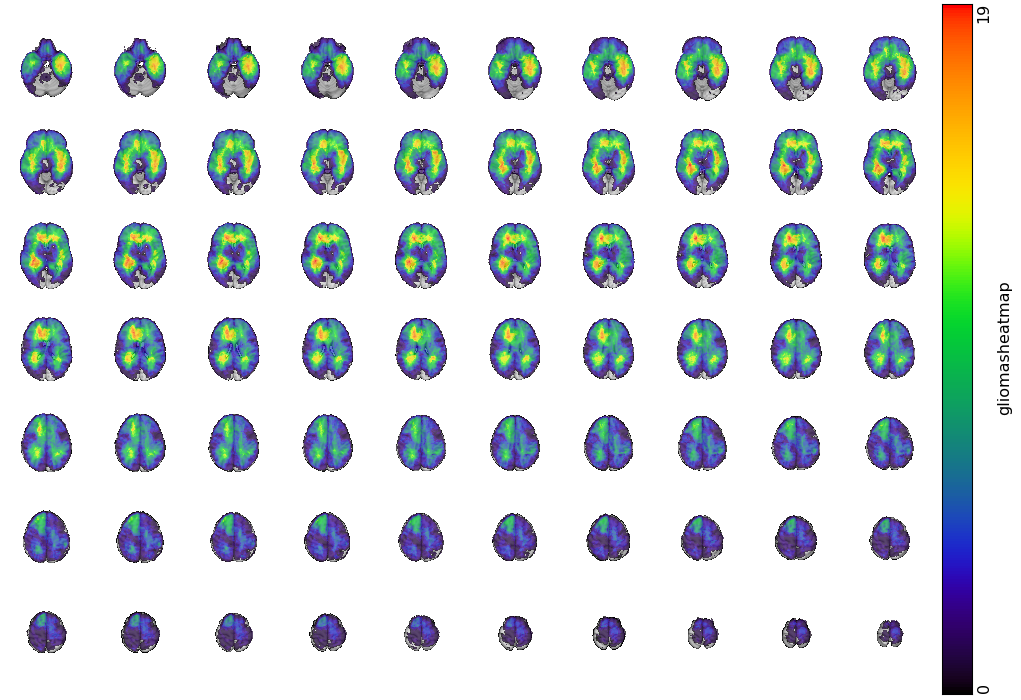


1 4


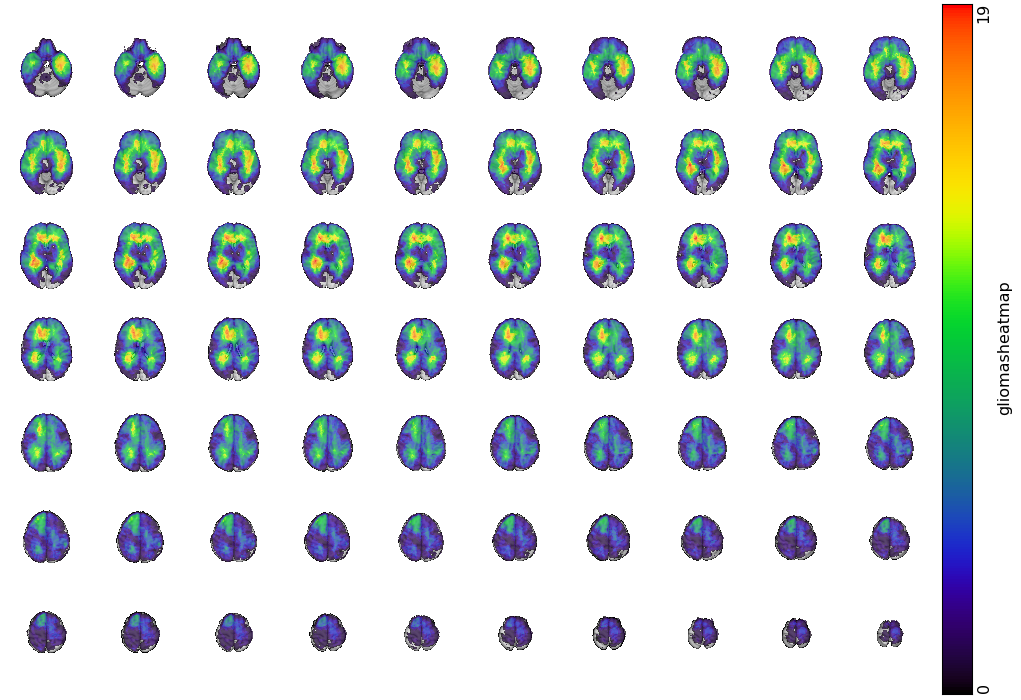


1 4

Supplementary Figure 3. Pituitary adenoma heatmap


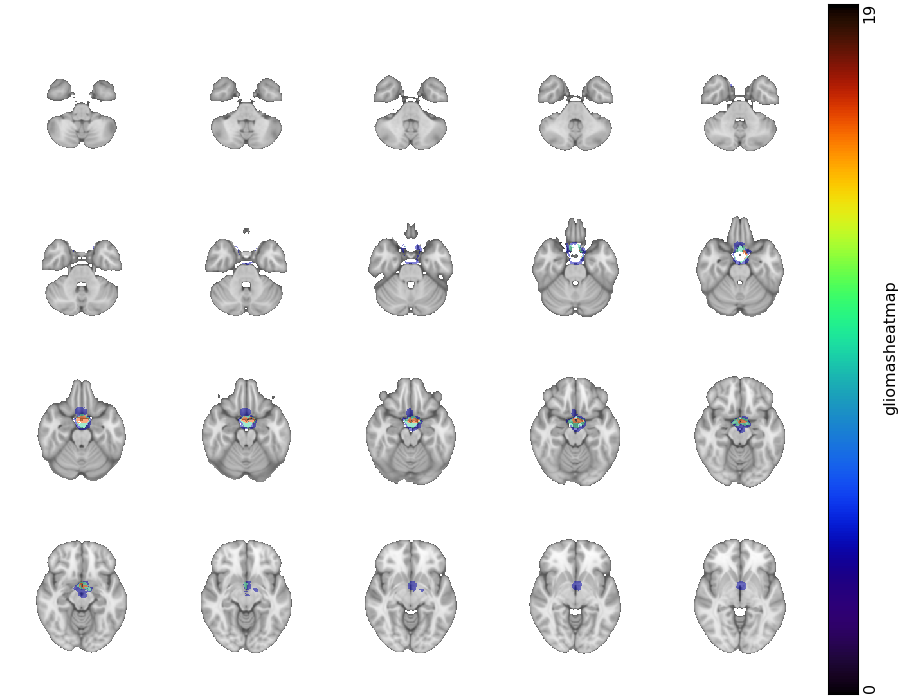


Supplementary Figure 4. Craniopharyngioma heatmap


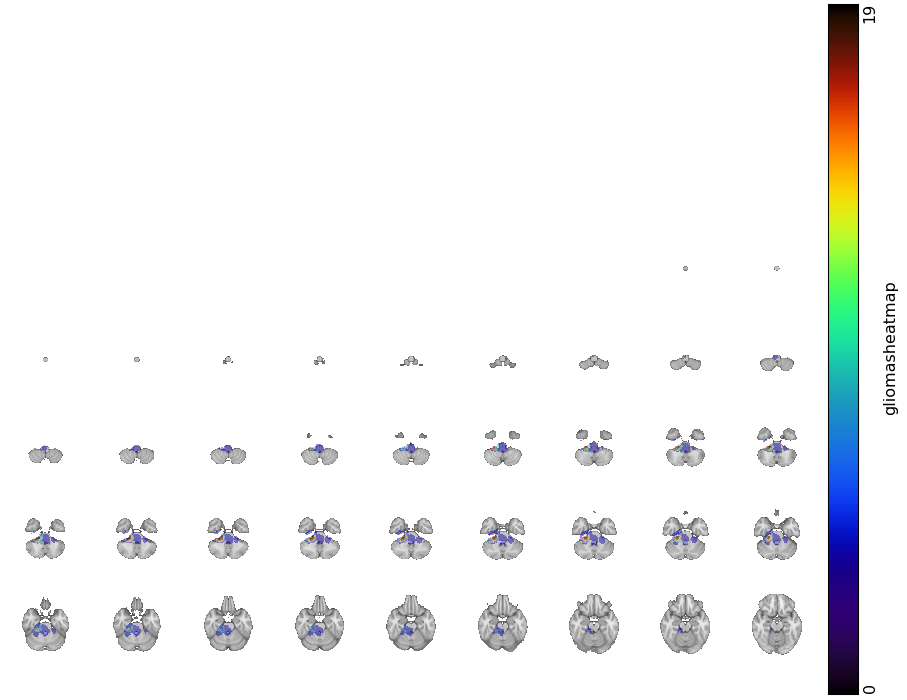


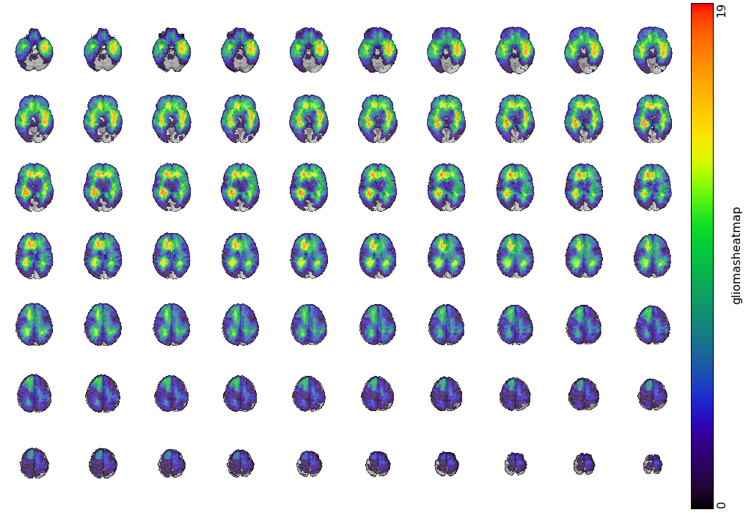


1 7

Supplementary Figure 5. Vestibular schwannoma heatmap

Supplementary Tables

**Table 1.** Prevalence numbers of impairment per tumor category

| **WHO grade** | **Impaired group** | **Not impaired group** | **Total group** |
| --- | --- | --- | --- |
| **COWA** |  |  |  |
| Craniopharyngiomas | 0 | 4 | 4 |
| Gliomas | 28 | 96 | 124 |
| Meningiomas | 4 | 24 | 28 |
| Pituitary adenomas | 1 | 8 | 9 |
| Vestibular schwannomas | 2 | 7 | 9 |
| Other | 1 | 3 | 4 |
| Total | 36 | 142 | 178 |
| **TMT A** |  |  |  |
| Craniopharyngiomas | 1 | 3 | 4 |
| Gliomas | 40 | 84 | 124 |
| Meningiomas | 4 | 24 | 28 |
| Pituitary adenomas | 0 | 9 | 9 |
| Vestibular schwannomas | 3 | 6 | 9 |
| Other | 0 | 4 | 4 |
| Total | 48 | 130 | 178 |
| **TMT B** |  |  |  |
| Craniopharyngiomas | 1 | 3 | 4 |
| Gliomas | 45 | 75 | 120 |
| Meningiomas | 5 | 23 | 28 |
| Pituitary adenomas | 0 | 8 | 8 |
| Vestibular schwannomas | 3 | 6 | 9 |
| Other | 1 | 3 | 4 |
| Total | 55 | 118 | 173 |
| **HVLT A** |  |  |  |
| Craniopharyngiomas | 0 | 4 | 4 |
| Gliomas | 29 | 96 | 125 |
| Meningiomas | 4 | 24 | 28 |
| Pituitary adenomas | 3 | 6 | 9 |
| Vestibular schwannomas | 2 | 7 | 9 |
| Other | 1 | 3 | 4 |
| Total | 39 | 140 | 179 |
| **HVLT B** |  |  |  |
| Craniopharyngiomas | 1 | 3 | 4 |
| Gliomas | 33 | 88 | 121 |
| Meningiomas | 3 | 25 | 28 |
| Pituitary adenomas | 4 | 5 | 9 |
| Vestibular schwannomas | 3 | 6 | 9 |
| Other | 2 | 2 | 4 |
| Total | 46 | 129 | 175 |

**Table 2.** Prevalence numbers of impairment in gliomas per surgery and WHO grade category

| **Surgery subgroups** | **Impaired group** | **Not impaired group** | **Total group** |
| --- | --- | --- | --- |
| **COWA** |  |  |  |
| No surgery | 1 | 10 | 11 |
| Biopsy | 11 | 25 | 36 |
| Resection | 16 | 60 | 76 |
| Total | 28 | 95 | 123 |
| **TMT A** |  |  |  |
| No surgery | 3 | 8 | 11 |
| Biopsy | 11 | 26 | 37 |
| Resection | 25 | 50 | 75 |
| Total | 39 | 84 | 123 |
| **TMT B** |  |  |  |
| No surgery | 4 | 6 | 10 |
| Biopsy | 14 | 22 | 36 |
| Resection | 26 | 47 | 73 |
| Total | 44 | 75 | 119 |
| **HVLT A** |  |  |  |
| No surgery | 2 | 9 | 11 |
| Biopsy | 12 | 25 | 37 |
| Resection | 14 | 62 | 76 |
| Total | 28 | 96 | 124 |
| **HVLT B** |  |  |  |
| No surgery | 3 | 7 | 10 |
| Biopsy | 11 | 25 | 36 |
| Resection | 18 | 56 | 74 |
| Total | 32 | 88 | 120 |
| **WHO grade** | **Impaired group** | **Not impaired group** | **Total group** |
| **COWA** |  |  |  |
| WHO 1 | 1 | 2 | 3 |
| WHO 2 | 8 | 27 | 35 |
| WHO 3 | 2 | 20 | 22 |
| WHO 4 | 17 | 45 | 62 |
| Total | 28 | 94 | 122 |
| **TMT A** |  |  |  |
| WHO 1 | 1 | 2 | 3 |
| WHO 2 | 5 | 29 | 34 |
| WHO 3 | 3 | 19 | 22 |
| WHO 4 | 30 | 33 | 63 |
| Total | 39 | 83 | 122 |
| **TMT B** |  |  |  |
| WHO 1 | 2 | 1 | 3 |
| WHO 2 | 7 | 27 | 34 |
| WHO 3 | 5 | 16 | 21 |
| WHO 4 | 30 | 30 | 60 |
| Total | 44 | 74 | 118 |
| **HVLT A** |  |  |  |
| WHO 1 | 0 | 3 | 3 |
| WHO 2 | 6 | 29 | 35 |
| WHO 3 | 3 | 19 | 22 |
| WHO 4 | 19 | 44 | 63 |
| Total | 28 | 95 | 123 |
| **HVLT B** |  |  |  |
| WHO 1 | 0 | 3 | 3 |
| WHO 2 | 4 | 30 | 34 |
| WHO 3 | 8 | 13 | 21 |
| WHO 4 | 20 | 41 | 61 |
| Total | 32 | 87 | 119 |

**Table 2.** Prevalence numbers of impairment in meningiomas per surgery and WHO grade category

| **Surgery subgroups** | **Impaired group** | **Not impaired group** | **Total group** |
| --- | --- | --- | --- |
| **COWA** |  |  |  |
| No surgery | 1 | 10 | 11 |
| Biopsy | 0 | 1 | 1 |
| Resection | 3 | 13 | 16 |
| Total | 4 | 24 | 28 |
| **TMT A** |  |  |  |
| No surgery | 2 | 9 | 11 |
| Biopsy | 0 | 1 | 1 |
| Resection | 2 | 14 | 16 |
| Total | 4 | 24 | 28 |
| **TMT B** |  |  |  |
| No surgery | 1 | 10 | 11 |
| Biopsy | 0 | 1 | 1 |
| Resection | 4 | 12 | 16 |
| Total | 5 | 23 | 28 |
| **HVLT A** |  |  |  |
| No surgery | 1 | 10 | 11 |
| Biopsy | 0 | 1 | 1 |
| Resection | 3 | 13 | 16 |
| Total | 4 | 24 | 28 |
| **HVLT B** |  |  |  |
| No surgery | 0 | 11 | 11 |
| Biopsy | 0 | 1 | 1 |
| Resection | 3 | 13 | 16 |
| Total | 3 | 25 | 28 |
| **WHO grade** | **Impaired group** | **Not impaired group** | **Total group** |
| **COWA** |  |  |  |
| WHO 1 | 1 | 14 | 15 |
| WHO 2 | 3 | 8 | 11 |
| WHO 3 | 0 | 2 | 2 |
| Total | 4 | 24 | 28 |
| **TMT A** |  |  |  |
| WHO 1 | 2 | 13 | 15 |
| WHO 2 | 2 | 9 | 11 |
| WHO 3 | 0 | 2 | 2 |
| Total | 4 | 24 | 28 |
| **TMT B** |  |  |  |
| WHO 1 | 1 | 14 | 15 |
| WHO 2 | 4 | 7 | 11 |
| WHO 3 | 0 | 2 | 2 |
| Total | 5 | 23 | 28 |
| **HVLT A** |  |  |  |
| WHO 1 | 2 | 13 | 15 |
| WHO 2 | 2 | 9 | 11 |
| WHO 3 | 0 | 2 | 2 |
| Total | 4 | 24 | 28 |
| **HVLT B** |  |  |  |
| WHO 1 | 1 | 14 | 15 |
| WHO 2 | 2 | 9 | 11 |
| WHO 3 | 0 | 2 | 2 |
| Total | 3 | 25 | 28 |
